# Identification of citrus diseases based on AMSR and MF-RANet

**DOI:** 10.1186/s13007-022-00945-4

**Published:** 2022-09-24

**Authors:** Ruoli Yang, Tingjing Liao, Peirui Zhao, Wenhua Zhou, Mingfang He, Liujun Li

**Affiliations:** 1 College of Computer and Information Engineering, Central South University of Forestry and Technology, Changsha, 410004 China; 2 College of Food Science and Engineering, Central South University of Forestry and Technology, Changsha, 410004 China; 3 Department of Civil, A-R-Chitectural and Environmental Engineering, University of Missouri-Rolla, Rolla, MO 65401 USA

**Keywords:** Citrus disease detection, AMSR, RAM, AugFPN, ELU activation function, MF-RANet

## Abstract

**Background:**

As one of the most widely planted fruit trees in southern China, citrus occupies an important position in the agriculture field and forestry economy in China. There are many kinds of citrus diseases. If citrus infected with diseases cannot be controlled in time, it easily seriously affects citrus production and causes large economic losses. Timely monitoring of disease characteristics in the citrus growth process is important for implementing timely control measures. Citrus images are easily disturbed by environmental factors such as dust, low light, clouds or leaf shadows. This makes it easy for some disease spot features in citrus pictures to be obscured. Occluded lesions cannot be effectively extracted and recognized. Second, similar characteristics of different diseases also make it difficult to distinguish the different types of diseases. However, the existing machine vision technology for identifying citrus diseases still has some difficulties in dealing with the above problems.

**Results:**

This paper proposes a new citrus disease identification framework. First, a citrus image enhancement algorithm based on the MSR-AMSR algorithm is proposed, which can enhance the image and highlight the disease characteristic information. The AMSR algorithm can also greatly alleviate the interference of clouds and low light on image lesions, making the image features clearer. Second, an MF-RANet network is proposed to recognize citrus disease images. MF-RANet is composed of a main feature frame and a detail feature frame. The main feature frame uses the cross stacking structure of ResNet50 and RAM to extract the main features in the citrus image dataset. RAM is used to extract the attention weight in the feature layer, which enables RAM to give higher weight to disease features. The detailed feature frame path uses AugFPN to extract features from multiple scales and fuse the main feature frame path. AugFPN enables the network to retain more detailed features, so it can effectively distinguish similar features in different diseases. In addition, we use the ELU activation function not only to solve the problem of gradient explosion and gradient disappearance but also to effectively use the negative input of the network. Finally, we use the label smoothing regularization method to prevent overfitting the network in the classification process. Finally, the experimental results show that the highest detection accuracy of the network for Huanglong disease, Corynespora blight of citrus, fat spot macular disease, citrus scab, citrus canker and healthy citrus is 96.77%, 96.22%, 95.96%, 95.93%, 94.04% and 97.55%, respectively.

**Conclusions:**

The citrus disease algorithm based on AMSR and MF-RANet can effectively perform the disease detection function. It has a high recognition rate for different kinds of citrus diseases. With the addition of AMSR preprocessing, RAM, AugFPN, ELU activation function and other structures, the MF-RANet network performance improves.

## Introduction

As one of the most widely planted fruit trees in southern China, citrus occupies an important position in the field of agriculture and forestry economy in China [1, 1]. With the continuous expansion of citrus market demand, its planting area is also increasing. This not only improves the citrus planting yield but also increases the probability of large-scale outbreaks of citrus diseases and economic losses [3]. There are many kinds of citrus diseases, and symptoms can appear in fruits, leaves, roots and other parts. Several disease symptoms may occur concurrently, which greatly increases the difficulty of judging the type of disease. At present, citrus disease diagnosis in China is based on the rich experience of fruit farmers. However, human judgement has problems such as strong subjectivity, large error, and difficulty unifying standards, and it is not easy to find early symptoms. Therefore, developing a reliable identification method for citrus diseases is conducive to taking effective measures to prevent and control diseases in time, which is of great significance to citrus production.

Traditional disease recognition technology is usually based on global features such as the colour, shape and texture of the disease spot and multiple indicators are selected to reflect the gap between the diseases. For example, Stegmayer et al. [4] used 10 characteristic parameters of colour, shape and texture to detect diseases such as citrus canker, black spot and citrus scab. However, this method has the disadvantages that it is difficult to distinguish different categories when the indicators cross and overlap, and the illumination intensity easily blurs the characteristics. Zhang et al. [5] proposed an apple defect detection method based on the fuzzy c-means algorithm and nonlinear programming genetic algorithm (FCM-NPGA) combined with multivariate image analysis. The FCM-NPGA algorithm is used to segment the suspicious defect image. This effectively detects the apple defect image. Zhang et al. [6] proposed a citrus surface defect classification method based on machine vision. They combined the improved convolution neural network with state transition algorithm (STA) to identify citrus surface defects. Compared with traditional disease identification technology, neural networks show obvious advantages in identification. An improved citrus surface defect recognition method based on convolution (STA) and neural networks was proposed. The discrimination rate of 1,000 defective and nondefective citrus images reached 99.1%. However, this method is limited to the recognition of defective and nondefective citrus images. It still shows obvious deficiencies in distinguishing citrus diseases. Mohanty et al. [7] constructed a method to recognize 26 plant disease images, including Huanglong disease images, based on a CNN and obtained a high overall recognition rate. The real environment is often different from the laboratory environment. The collected images are easily affected by the surrounding environment, which affects the recognition accuracy of the network. Lin et al. [8] proposed a banana disease recognition model based on EM-ERNet, which improved the adaptability of the model to banana disease image samples. However, different kinds of diseases also have similar characteristics, and the characteristics are more difficult to distinguish due to the influence of image quality. The citrus symptoms in the early stage are not obvious, and there may still be problems that make it difficult to collect detailed features. Therefore, the main problems in recognizing citrus diseases are as follows. (1) The characteristics of citrus lesions in the image are not clear. In the real environment, the disease spots in citrus images are easily affected by dust, clouds or leaf shadows. When the ambient brightness is low, it is difficult to recognize and extract citrus lesions where the local brightness is too low in the citrus image. The above reasons lead to the network not being able to extract the complete disease information and reduce the accuracy of citrus disease recognition. (2) There are similarities among different kinds of citrus disease spots. If similar lesions want to be accurately classified, it requires the network to extract detailed features with differences between different types. Detailed features are often easily lost in the extraction process, which easily affects the citrus disease recognition accuracy on the network.

To solve the problem that the disease features in the image are vulnerable to environmental interference, LV et al. [9] used pulse coupled neural network (PCNN) image segmentation method based on minimum cross entropy to segment apple image. The recognition accuracy of Apple disease after segmentation is 93%. Zhang et al. [10] segmented the lesion and extracted the color, shape and texture features of the lesion. Then, the k-nearest neighbor (KNN) classification algorithm is used to identify five kinds of corn leaves, and the recognition accuracy is more than 90%. Zhou et al. [11] proposed a fast rice disease detection method based on the fusion of FCM-KM and fast R-CNN to solve the problems of noise, blurred edges, large background interference and low detection accuracy in rice disease images. A two-dimensional filter mask combined with a weighted multistage median filter (2DFM-AMMF) is used for noise reduction, and a faster two-dimensional Otsu threshold segmentation algorithm is used to reduce the interference of complex backgrounds on target leaf detection in the image. Chen et al. [12] used the binary wavelet transform and retinex algorithm to enhance and denoise the image, but the above two methods lose some overall image features when denoising and extracting important texture information in the target object.

Therefore, this paper proposes the AMSR algorithm to enhance citrus disease images. The AMSR algorithm is improved based on the traditional MSR [13] algorithm. Therefore, the AMSR algorithm inherits the advantages of the traditional MSR algorithm. It can not only enhance the overall brightness of the image but also has denoising ability. The AMSR algorithm removes some noise points generated by the equipment and environmental noise, such as clouds and dust, in citrus disease images. Second, the uneven brightness distribution of the target in the image can be compensated by introducing the brightness factor in the AMSR algorithm. Therefore, the AMSR algorithm can also realize local illumination in places with low local illumination in the image.

To solve the problem that the feature similarity and detail feature loss of citrus diseases affect neural network recognition, Sankaran et al. [14] used the massive accumulation effect of Huanglong disease leaves on starch. They used mid-range infrared spectroscopy to detect Huanglong disease. The classification accuracy of Huanglong diseased leaves, healthy leaves and deficient diseased leaves reached more than 90%. However, the process of hyperspectral data acquisition is complex, and this method has difficulty meeting the requirements of orchard operations in practice. Liu et al. [15] proposed a real-time detection method of tomato grey spot under complex background. This method can effectively extract the early characteristics of tomato grey spot. Chen et al. [16] established a model for fruit classification based on a multiple optimized convolution neural network. The optimized convolutional neural network has a high recognition rate for fruit classification. The data acquisition process of deep neural networks is relatively simple, and it shows advantages in some visual recognition tasks. However, when the network layer is too deep, disappearing or exploding gradient occurs. LV [17] and others used the PReLU activation function and AdaBound optimizer to improve convergence and accuracy when identifying corn leaf diseases. However, they do not have strong anti-interference ability, and the network has a poor cognitive effect on some similar fine-grained features of citrus diseases.

Therefore, aiming at the problem that there are similar characteristics among different citrus disease species and the details are easily lost, this paper constructs an MF-RANet model. The MF-RANet model contains two frame paths for feature extraction: the main feature frame path and the detail feature frame path. The main feature frame takes ResNet50 as the main structure of the network and adds the attention mechanism [18–20]—RAM to extract the fine-grained features of the citrus diseases image. The detail feature frame uses the AugFPN feature fusion algorithm, which can preserve some detail features while deepening the network. In addition, we use the ELU activation function not only to solve the problem of gradient explosion and gradient disappearance but also to effectively use the negative input part of the network. Finally, we use the label smoothing regularization method in the classification output of the softmax function to prevent overfitting the network during classification. In the implementation process of this paper, the main contributions are as follows: a new citrus image enhancement algorithm, AMSR, is proposed. The AMSR algorithm adopts a Gaussian filter and the retinex [21] algorithm to realize three-channel global enhancement for the three colour channels after image decomposition. Second, the three enhanced colour channels are weighted and combined according to the frequency difference in the image. Finally, the brightness compensation factor is introduced for local and detail enhancement.

1. To prevent the loss of detailed features and extract similar features among different citrus diseases, a new MF-RANet network is proposed to identify citrus diseases. The design is as follows:
2. We use RAM for MF-RANet. RAM cross cascades the 1– 4 layers of ResNe50 with RAM blocks with the same residual structure, which can realize the residual structure with high depth and high width. This residual structure is conducive to extracting important features.
3. We use the AugFPN feature fusion algorithm for MF-RANet. AugFPN can effectively reduce the loss of small citrus disease features by extracting and fusing features of multiple scales. The AugFPN structure and ResNet50 extract features together and can jointly realize size feature extraction.
4. In MF-RANet network, we use ELU activation function instead of ReLU activation function. The ELU activation function can not only solve the problems of gradient explosion and gradient disappearance in the network but also effectively use the negative input part of the network. Second, the effective activation brought by the ELU activation function can improve the network citrus disease recognition rate and convergence speed.
5. We add label smoothing regularization after the softmax function of MF-RANet network. Label smoothing regularization can effectively prevent overfitting in the label smoothing network classification process. Introducing label smoothing regularization can effectively improve citrus disease recognition network accuracy.
6. The average test recognition accuracy of the six citrus pictures was 96.87%, and the F1 score was 96.56%. This method not only has a good effect on identifying citrus health and disease but also has a good effect on classifying citrus diseases with similar disease spots. The method in this paper can also be used in public datasets with good effect. It can reduce the agricultural citrus loss due to disease. Rapid and accurate identification and classification of diseases can effectively reduce the loss of citrus to diseases in agricultural production.

**Fig. 1:**
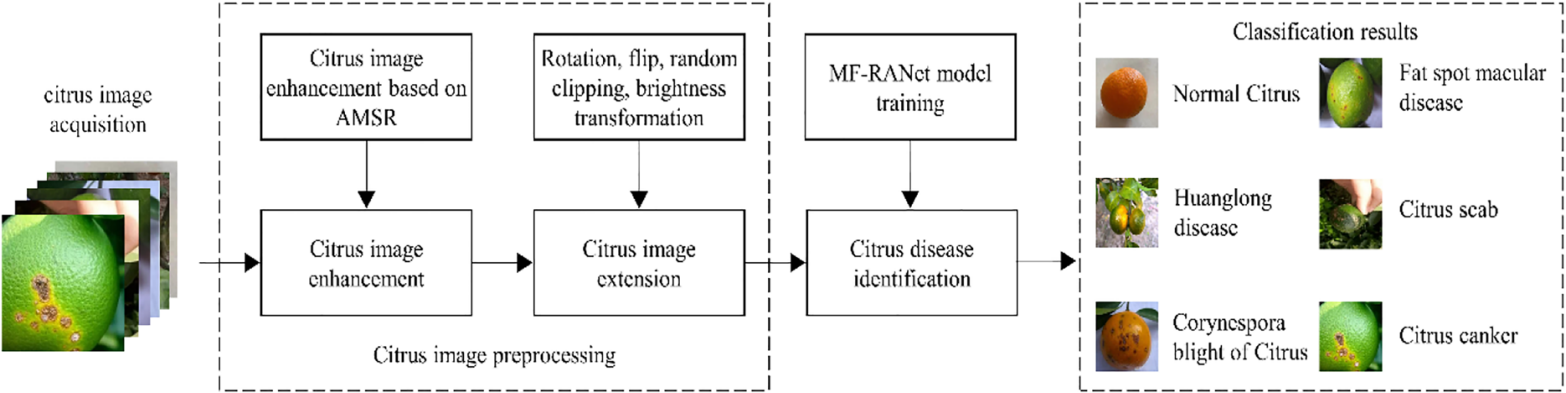
Schematic diagram of citrus diseases identification

A schematic diagram of citrus disease recognition is shown in Fig. 1 Schematic diagram of citrus diseases identification. First, the citrus disease image is enhanced through image preprocessing and the image is highlighted globally. Details and local enhancement are realized at low local illumination or with shadows caused by clouds, dust and leaves. It can effectively reduce the interference of low illumination, clouds, dust and leaf occlusion on the disease characteristics in the image. Second, the enhanced citrus disease image is widened. Finally, the amplified dataset is used to identify and classify citrus diseases through the MF-RANet network.

## Materials and methods

### Data acquisition

The dataset used in the experiment comes from 2 sources: the dataset website and orchard collection. The dataset website includes the China Science Data Network [22] and digipathos Website [23]. A total of 736 images of 5 common citrus diseases were carefully selected on the dataset website. The other source is in cooperation with Central South University of Forestry Science and Technology. The image data were collected from the economic forest and fruit production, study and research base jointly built by Central South University of Forestry Science and Technology and Changsha Forestry Bureau. The camera model is a Canon EOSR, and its image pixels are 2400*1600. We used a camera to take optical images of different diseases and normal citrus from different angles in the morning, middle and evening under sunny, cloudy and foggy weather conditions. The shooting background is the complex background of the orchard. Such photos can reflect many complex citrus growth situations in the orchard to ensure that the collected images are more representative. A total of 1772 images were finally collected, including 482 samples with uniform illumination on sunny days, 531 samples with uneven illumination and 368 samples on cloudy days. A total of 391 samples were disturbed by clouds and dust. A total of 2525 images of five diseases and normal citrus were finally obtained through orchard collection and dataset websites. There are 687 samples with uniform illumination in sunny days, 534 samples in cloudy days and 757 samples with uneven illumination. 557 samples disturbed by clouds and dust. Because a large number of datasets are needed for network training, we enhanced the original citrus image and amplified the data by rotating, flipping, random clipping and brightness transformation. A total of 10,100 images were finally obtained in the database. Table 1 shows the categories and data distribution of citrus diseases selected in this paper, including healthy citrus, Huanglong disease, citrus Corynespora, citrus fat spot yellow spot, citrus scab and citrus canker.

**Table 1:** Image quantity distribution of six citrus diseases

Disease rate	Example	Number	Proportion (%)	Weather conditions of the picture			
				Sunny	Cloudy	Foggy	Uneven illumination
Normal citrus		1624	16.08	444	335	490	355
Huanglong disease		1696	16.79	459	353	509	375
Corynespora mildew		1891	18.72	510	401	557	423
Fat spot macular disease		1694	16.77	458	353	508	375
Citrus scab		1556	15.41	427	318	471	340
Citrus canker		1639	16.23	450	336	493	360

### Citrus image enhancement based on AMSR

A variety of citrus plants are planted in mountainous and hilly areas, which are prone to cloudy and rainy weather. In the process of collecting citrus disease images, the images may be disturbed by dust, clouds, low light and other environments. This may result in some lesions in the dataset being blocked or unclear. For example, in Fig. 3, citrus yellow dragon disease with fog and Corynespora blight on citrus with low light. The disease characteristics of some citrus were not obvious under low light and cloud occlusion. Due to the limitation of the citrus environment, the network citrus diseases identification accuracy is reduced to a certain extent. To help further improve citrus disease recognition accuracy in the follow-up network, this paper uses the AMSR algorithm to enhance citrus disease images. The AMSR algorithm can effectively alleviate the interference of clouds and low light on the clarity of citrus disease spots. Figure 3 shows that after image enhancement, the disease spot features in the citrus Huanglong image obscured by clouds and the Corynespora brightness of the citrus image in low light become clear and obvious. This is more conducive to the neural network for extracting the characteristics of subsequent citrus diseases.

**Fig. 2:**
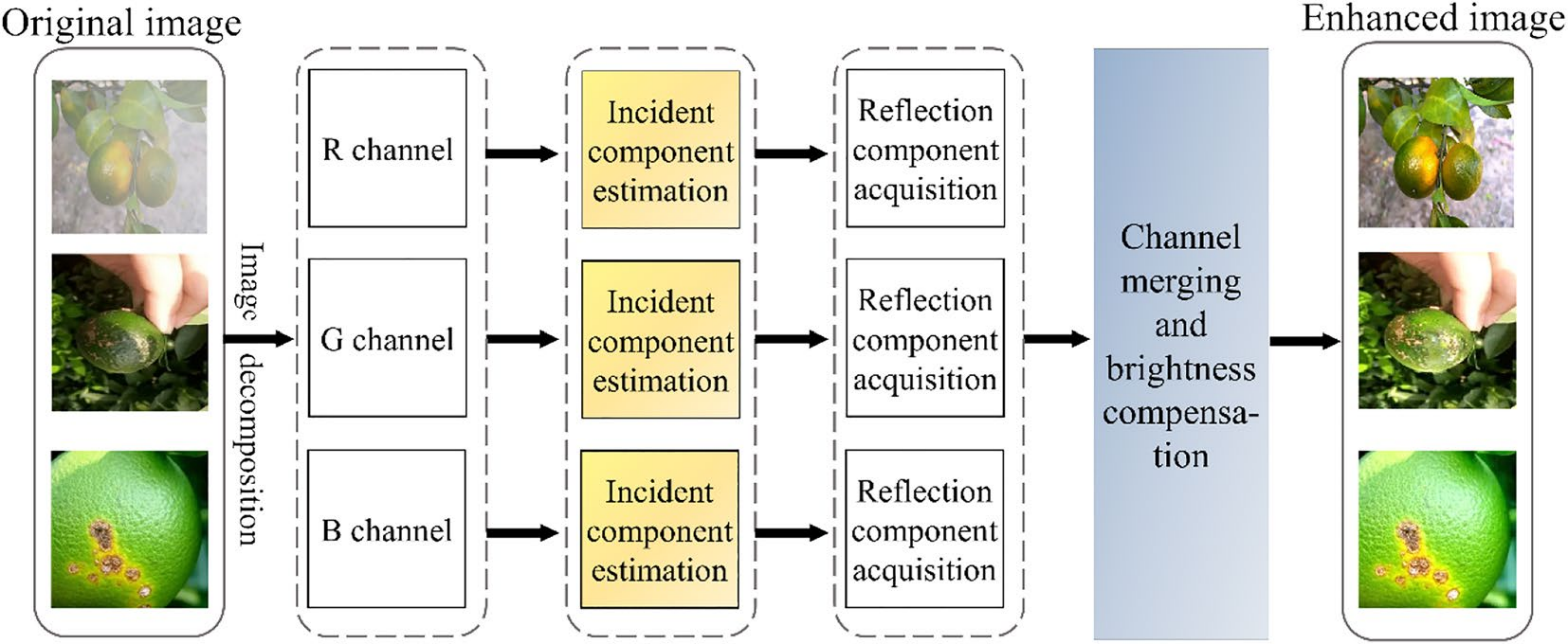
AMSR algorithm block diagram

In citrus image enhancement based on ASMR, first, the original image of citrus diseases is decomposed to obtain three RGB colour channels. Second, the incident component of a single channel is estimated, and three Gaussian surround functions are constructed by using three scale parameters. The Gaussian surround function is used to filter the image channels, and then the weight coefficient is introduced to obtain the three-channel incident component weight of each region. Next, the reflection component is calculated. The reflection component is obtained by subtracting the original image from the illumination component in the logarithmic domain. Finally, the R, G and B channels of the whole picture are restored, and the brightness compensation factor is added to repair and adjust the defect of colour distortion caused by contrast enhancement in local areas of the image. The schematic diagram of the AMSR algorithm is shown in Fig. 2, in which the original input image Fig. 2a is the citrus yellow dragon disease image taken on cloudy days, Fig. 2b is the citrus scab image with uneven illumination on sunny days, and Fig. 2c is the citrus ulcer disease image with uniform illumination on sunny days. Finally, the enhanced images in three different cases are obtained by the AMSR algorithm. The operation process of the AMSR algorithm is divided into the following steps:

### Image decomposition

The resulting citrus disease image is decomposed into three colour channels: R, G and B. Subsequent calculations are implemented in each channel.

### Incident component estimation

#### (1) Single-channel incident component estimation

The incident component reduces the high-frequency difference of the original fluctuating citrus diseases image, covers the disease features, blurs the edge details, and visually becomes a large black (homogeneous area). Due to the influence of incident light, the resulting picture deviates from the inherent properties of the object, so it is necessary to eliminate the interference of incident light. Next, the Gaussian surround function is used to filter the three channels of the citrus disease image to estimate the incident light. The three-channel Gaussian surround function can be expressed by the following formula:

1 $${\text{G}}\left( {x,y} \right) = \frac{1}{{2\pi \sigma^{2} }}e^{{\left( { - \frac{{x^{2} + y^{2} }}{{2\sigma^{2} }}} \right)}}$$

In the formula, σ is the Gaussian surround scale, which is related to the overall smoothness of the Gaussian function. The incoming and outgoing images are calculated by Fourier transform.

#### (2) Weight analysis of three channels

Three different weights represent different incident components in different regions. Because different pixels are located in different regions, they have different weights. The pixel weight is determined by judging whether the pixel is in the image edge area carrying high-frequency information or in the homogeneous area carrying low-frequency information. If the pixel is in the edge area (carrying high-frequency information), the effect image with a smaller $\upsigma$ value has greater weight. If the pixels are in the homogeneous area (carrying low-frequency information), the rendering with a larger $\upsigma$ value has greater weight. This article presents a new weight coefficient, the frequency comparison coefficient, which is calculated as follows:

2 $$A = a_{1} \cos^{ - 1} \left( \sigma \right) + a_{2}$$

$\upsigma$ is the standard deviation of the local area where the pixel is located. When the standard deviation of a region is small, the pixel value fluctuation in the region is very small, which means that the region where the pixel is located belongs to the homogeneous region. The $arccot$ function is applied to $\upsigma$ to make the value in the homogeneous area larger, that is, the part with lower brightness information in the homogeneous area is taken into account to make the image information more clearly visible. In contrast, the value in the edge detail area is smaller. The estimated three channel reflection image can be obtained by adding their respective weights to the filtering results obtained on the three scales.

### Acquisition of the reflection component

A given citrus disease image S (x, y) can be decomposed into two different images: reflected image R(x, y) and incident image L (x, y). The incident light is reflected on the reflected object and is reflected into the human eye after reflection of the object. The final formed image can be represented by the following formula:

3 $${\text{S}}\left( {x,y} \right){\text{ = R}}\left( {x,y} \right){\text{*L}}\left( {x,y} \right)$$

where S (x, y) represents the image taken by the camera, L (x, y) represents the irradiation component of external light, R (x, y) represents the reflection component of the photographed object, and * represents simple multiplication. Then, a logarithmic operation is performed on the above formula, and the formula is as follows:

4 $${\text{Log}}\left[ {R\left( {x,y} \right)} \right] = {\text{Log}}\left[ {S\left( {x,y} \right)} \right] - Log\left[ {L\left( {x,y} \right)} \right]$$

L (x, y) is approximately replaced by the convolution of S (x, y) and a Gaussian kernel. L (x, y) is the incident component estimated above; then, R (x, y) can be expressed by the following formula:

5 $$R_{{MSR_{i} }} = \sum\nolimits_{n = 1}^{N} {\omega_{n} R_{ni} } = \sum\nolimits_{n = 1}^{N} {\omega_{n} } \left\{ {{\text{log}} S_{i} \left( {x,y} \right) - {\text{log}}\left[ {G\left( {x,y} \right)*S_{i} \left( {x,y} \right)} \right]} \right\}$$

In the above formula, S is the original input image, F is the Gaussian filter function, N is the number of scales, $\omega$ is the weight of each scale, and R represents the output of the image in the log domain. Since the incident component L (x, y) is obtained by Gaussian convolution, the reflected component can be obtained by using the above formula. Then, the weights of the filtered results on the 3 scales are added to obtain the estimated illuminance image. ${\omega }_{k}$ represents the weighting coefficient when the k-th scale is weighted, which needs to be met:

6 $$\sum\nolimits_{n = 1}^{N} {\omega_{k} } = 1$$

### Channel merging and brightness compensation

The grey reflection components are combined and brightness compensated to restore the three colour channels of R, G and B, and the increased brightness compensation factor is $\lambda$. The formula is as follows:

7 $$R_{j} \left( {x,y} \right) = \frac{{I_{j} \left( {x,y} \right)}}{{I\left( {x,y} \right)}}*\lambda$$

${I}_{j}(x,y)$ refers to the R, G and B channels of the original image, and $\lambda$ refers to adjusting the brightness factors of the three bands. Through experiments, it is found that the effect of $\lambda$ taking 1 is better. The AMSR algorithm proposed in this article can enhance and preserve the edge information under low illumination based on ensuring the image colour, and the principle of the algorithm is simple. The AMSR algorithm can solve the contradiction that details and picture colours cannot be retained simultaneously, and its actual effect on image enhancement is significantly improved compared with the MSR algorithm.

**Fig. 3:**
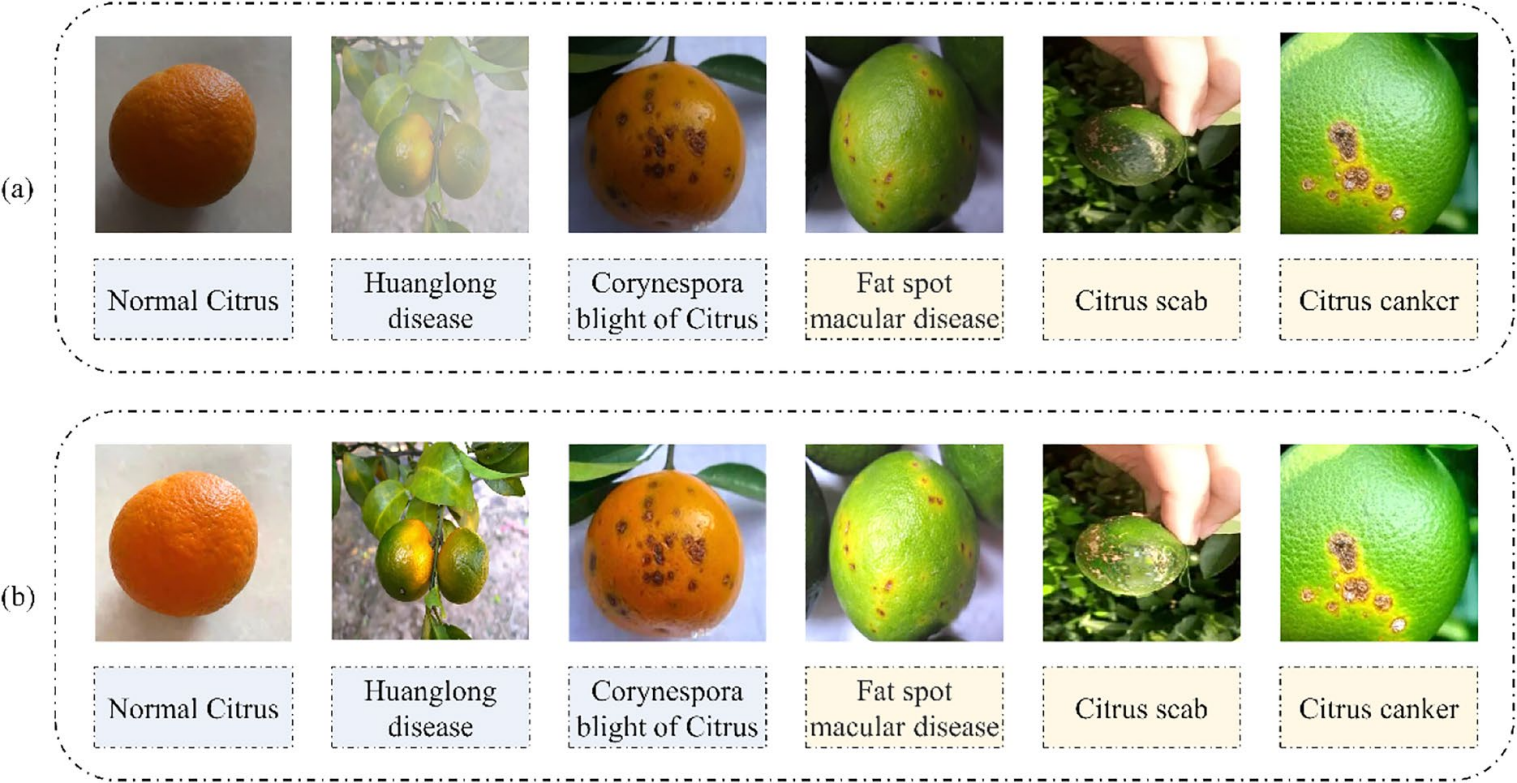
Comparison of citrus disease images before and after enhancement

The comparison of citrus disease images in the AMSR enhancement algorithm before and after enhancement is shown in Fig. 3. Figure 3a shows the actual images of five diseases and healthy citrus taken in three scenarios: cloudy, even and uneven light on sunny days; Fig. 3b is the citrus image enhanced by AMSR:

### Identification of citrus diseases based on MF-RANet

Many kinds of citrus diseases have similar characteristics. For example, both corynespora blight of citrus and fat spot macular disease in Fig. 3 have dark brown scar-like concave small particles, and a few are yellow–brown spots. Yellow white upwards convex lesions were found in both citrus scab and citrus canker disease, and their distribution positions were relatively concentrated. There are also different imaging features in the early and late stages of the same disease. The location of early diseases is hidden, and the area is small. Therefore, recognizing citrus diseases is difficult and requires the use of a deep neural network to extract more detailed feature recognition to achieve a higher degree of recognition. When using a deep neural network for feature extraction, the network deepens after reaching a certain depth, and the possibility of gradient degradation increases, which does not improve the classification performance. This leads to slower network convergence, lower accuracy, and easy loss of the main features. At this time, even if the dataset is increased, the classification performance and accuracy will not be improved. Therefore, we propose a new network structure MF-RANet to solve the above problems. MF-RANet is composed of a main feature frame path and a detail feature frame path. They extract the main recognition features and detailed features in citrus diseases.

**Fig. 4:**
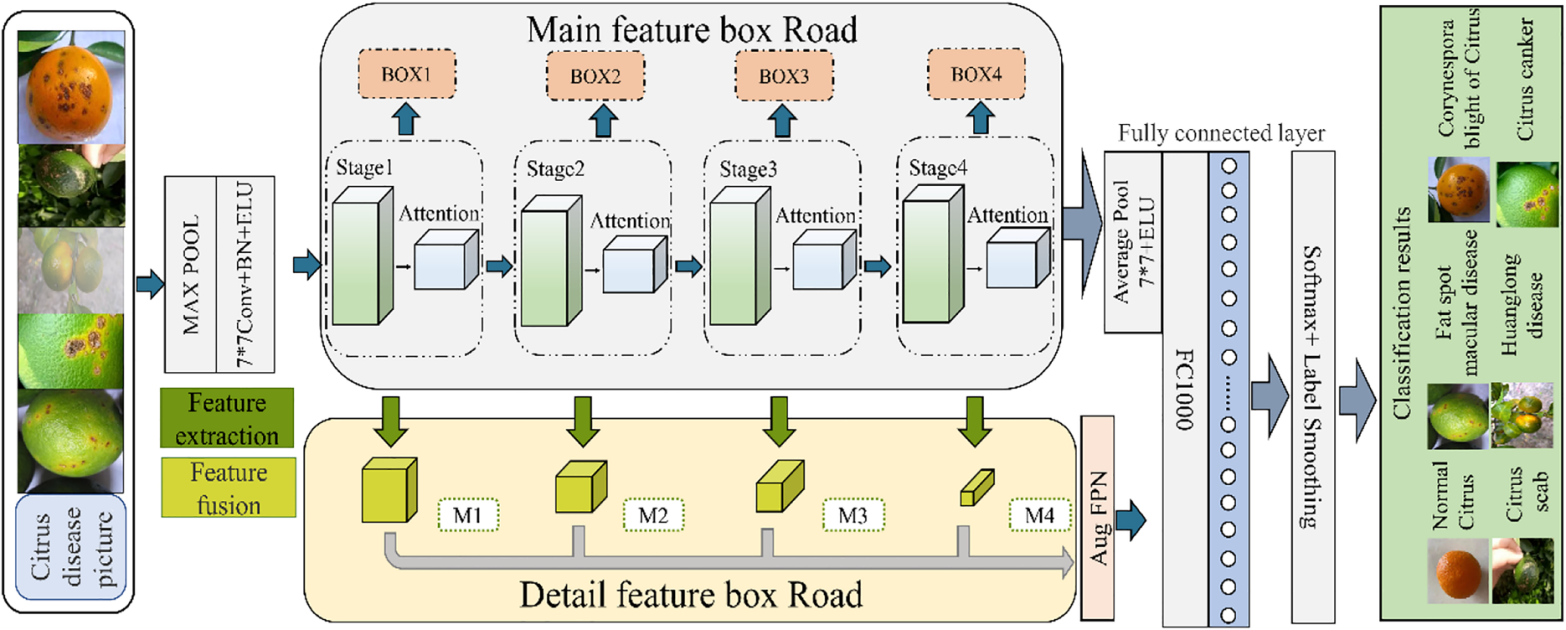
Schematic diagram of the MF-RANet model

The MF-RANet overall model built in this article is shown in Fig. 4. In the figure, the main feature frame path takes the ResNet50 network as the main body, and layers 1–4 in the ResNet50 network are represented as stages 1–4 in the figure. First, the attention mechanism is used to focus attention on some important or interesting information. Then, each layer of the ResNet50 network is cross stacked with four attention modules, and the resulting structure is Boxes 1–4 in Fig. 4. The resulting structure gives different weights to the feature information processed in the ResNet50 network to filter out the unimportant information and extract the large available features required for the identification of citrus diseases. This process can effectively improve processing efficiency and model performance. In addition, the detailed feature frame path extracts features in parallel with the main feature frame path in the graph, and all lead to the full connection layer. A feature fusion module is added on each layer of the main feature frame path in parallel. The micro features in each layer are extracted to obtain the detail feature layers of layers M1–4 in Fig. 4. Finally, the detailed features fused in layers M1–4 are input to the whole company layer and detected together with the large information features. This avoids the loss of small features to a great extent and optimizes the network performance. The experimental results show that the network structure composed of the main feature frame and detail feature frame plays an important role in citrus disease recognition accuracy. In addition, we use the ELU activation function in the basic ResNet50 network to solve the problems of gradient disappearance and gradient explosion. At the end of the network, we use label smoothing regularization to suppress the overfitting of the MF-RANet network and improve citrus disease classification accuracy.

### Main feature box road

The main feature box is composed of each layer of the ResNet50 network and attention module. The following focuses on the ResNet50 and RAM structures.

#### 1. ResNet50

ResNet is a network model proposed by He et al. [24] in 2015. At present, it has surpassed a series of algorithms, such as VGG [25], R-CNN [26], Fast R-CNN [27], and Faster R-CNN [28], in image classification and has become a basic feature extraction network in the field of general computer vision. ResNet50 uses a residual unit, which reduces the number of parameters and adds a direct channel in the network, increasing CNN's ability to learn features [29]. It can solve the difficult problem of gradient vanishing network training in a deep network. Through this residual unit structure, the network learning goal can be simplified, and the classification accuracy can be improved, which has good portability.

Each layer of the ResNet50 network contains 2 modules, an identity block and a convolution block. Convolution blocks can change the network dimension, but they cannot be connected in series; identity blocks are used to deepen the network and can be connected in series. With the deepening of the network level, the learned things become more complex, and the number of output channels increases. Therefore, while using identity blocks to deepen the network, it is also necessary to use convolution blocks to convert dimensions so that the features of the part in front can be transmitted to the feature layer in the back. Compared with previous networks, it is still one of the classic and used networks because of its few parameters, deep layers and excellent classification and recognition effect. However, for the problems that small features are easy to ignore and similar features are not easy to distinguish in the identification of citrus diseases, a single ResNet50 structure is still not enough. Therefore, this article improves upon ResNet50.

#### 2. RAM

**Fig. 5:**
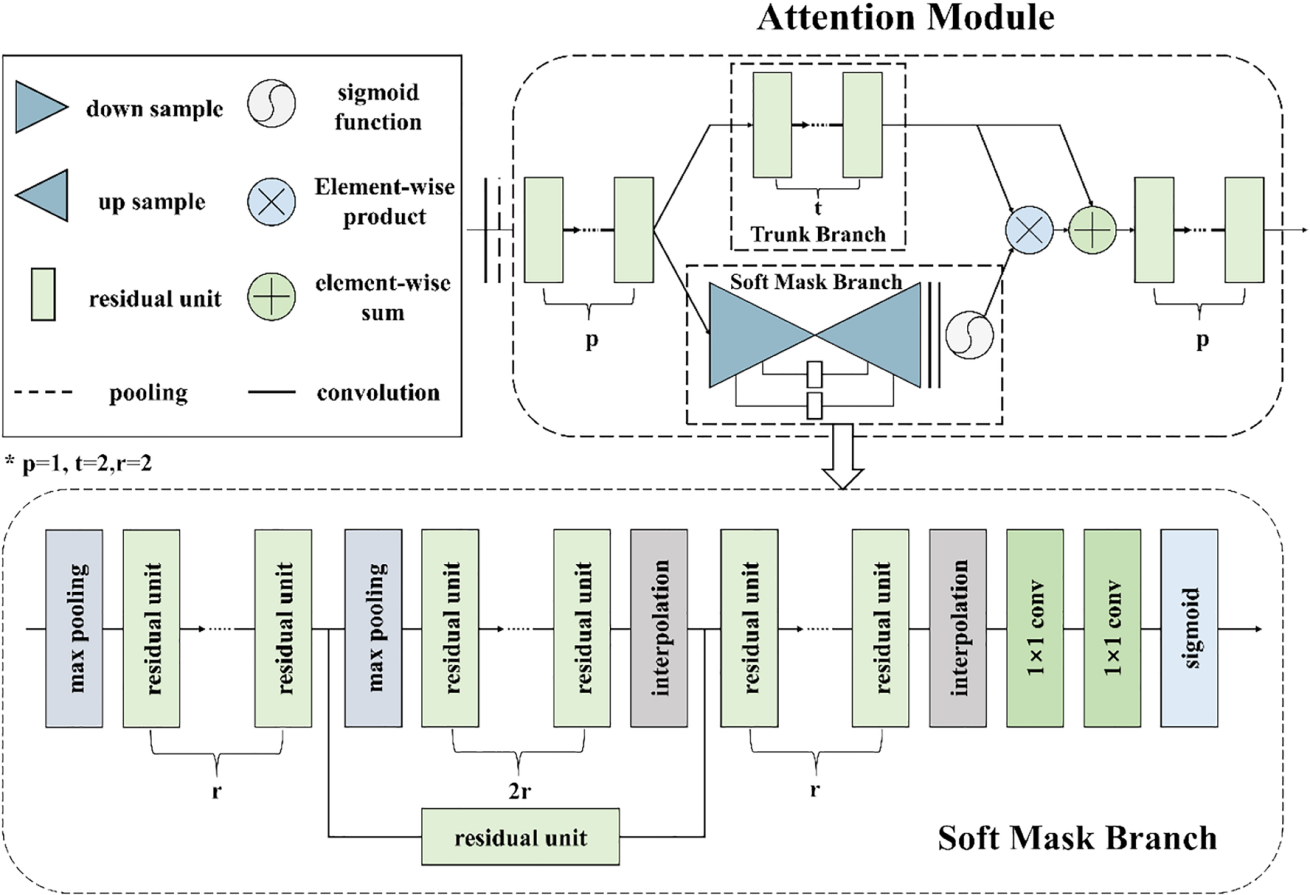
RAM schematic diagram

When people observe and recognize the target, they will focus on the prominent part of the target and ignore some global and background information. This selective attention mechanism is consistent with the characteristics of the discrimination part in fine-grained image classification. Then, in order to focus on monitoring the different features of the citrus diseases image, this paper cross adds the residual attention mechanism in each layer of resnet50. The RAM can give higher weight distribution to the features containing disease identification information. It can effectively improve the effect of fine-grained classification. There are two branches in RAM, namely, the mask branch and trunk branch. The trunk branch is convolution, and the mask branch outputs the attention feature map with the same dimension through feature map processing. Then, the characteristic graphs of the 2 branches are combined by point multiplication to obtain the final output characteristic graph. Finally, the RAM model is formed, as shown in Fig. 5. The structure and model construction of the RAM module are introduced below:

In the trunk branch structure, there are two convolutions in the main RAM branch structure, and the input features are directly processed into the same size as the mask branch structure 7 × 7.

In the mask branch structure, the processing operation of the feature map includes a forward downsampling process and an upsampling process. The downsampling process ensures fast coding and obtains the global features of the feature map. Upsampling combines the extracted global high-dimensional features after upsampling with the features without downsampling to fuse the features of high and low latitudes. The specific operations are as follows: mask branch for fixed input, after multilayer convolution calculation, use maximum pooling to reduce the feature map dimension. The dimension is reduced until the width and height of the feature map reach the minimum size of the network output feature map 7 × 7. Then, the width and height dimensions of the feature graph are expanded layer by layer by using the bilinear difference method and added to the previous features under the same dimension. The mask branching structure combines global and local features to enhance the expression ability of the feature map.

The RAM model built from these 2 parts is described below. The trunk branch output characteristic diagram is ${T}_{i,c}(x)$. The output characteristic diagram of the mask branch is ${M}_{i,c}\left(x\right)$. Finally, the output characteristic diagram of the attention module is ${H}_{i,c}\left(x\right)$; the framework formula of the model is:

8 $$H_{i,c} \left( x \right) = \left[ {1 + M_{i,c} \left( x \right)} \right]*T_{i,c} \left( x \right)$$

${M}_{i,c}\left(x\right)$ is the value in the [0,1] interval. Adding them to 1 can well solve the problem of reducing eigenvalues proposed in 1. In this part, the difference between this article and the residual network is that the formula ${H}_{i,c}\left(x\right)$=x+${T}_{i,c}(x)$ of the residual network learns the residual result between output and input, while in this article, ${T}_{i,c}(x)$ is learned and fitted by a deep convolutional neural network structure. Combined with the results of the mask branch output, the important features in the output characteristic diagram of ${T}_{i,c}(x)$ can be strengthened, while the unimportant features can be suppressed. Finally, the overlapping residual attention module and the residual block of ResNet50 can gradually improve the expression ability of the network.

### Detailed feature box road (AugFPN)

**Fig. 6:**
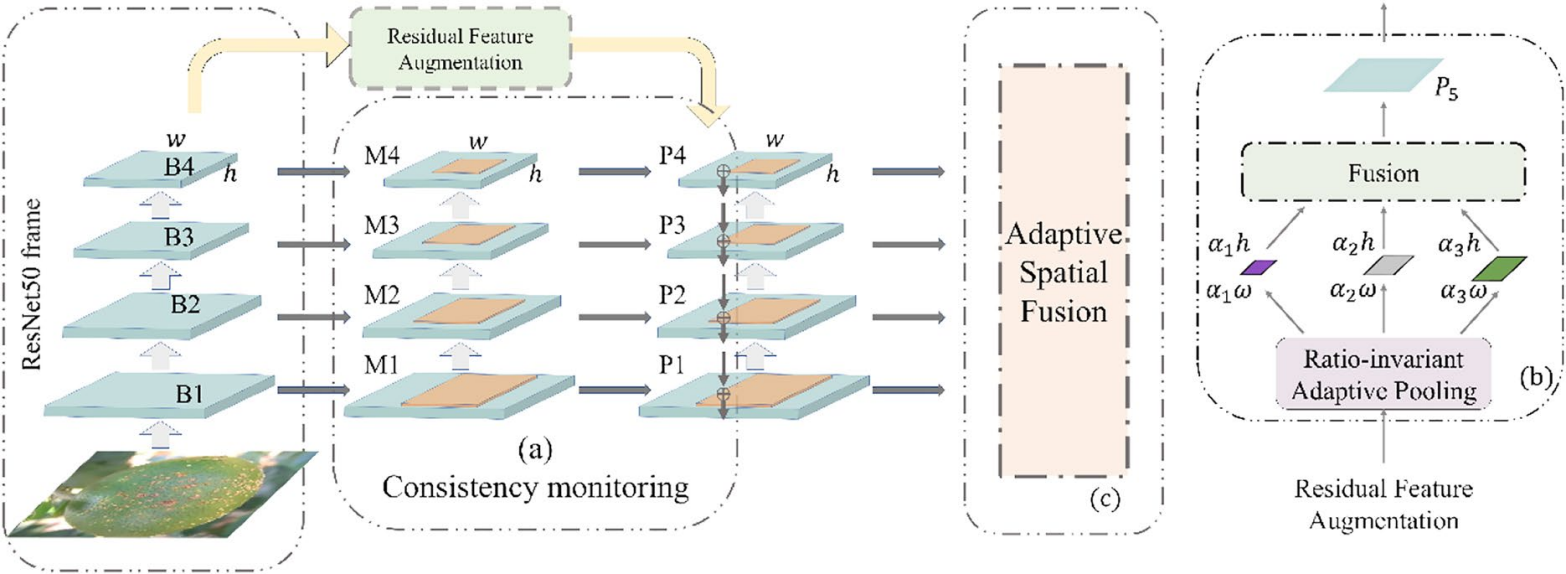
Schematic diagram of the AugFPN fusion framework

The detail feature box is used to extract the detail features by AugFPN feature fusion. As mentioned above, a network for identifying citrus diseases is proposed based on ResNet50. Although the features are extracted by convolution, after resampling again, some small pixel object features have been lost and cannot be recognized effectively. To ensure that the detailed features are not lost in the citrus disease recognition, the object features of any size can be effectively detected, and the correct recognition results can be obtained. Based on the main feature frame path, this article uses the AugFPN after the improved FPN [30] to add feature fusion. First, the consistency monitoring mechanism is used to implement the same monitoring signal on these feature maps so that the laterally connected feature maps contain similar semantic information. Second, the residual features are enhanced, and the ratio invariant adaptive pool is used to extract different context information to reduce the information loss of the highest level features in the feature pyramid by means of residuals. Third, soft ROI selection is introduced to make better use of ROI features at different pyramid levels to provide better ROI features for subsequent location refinement and classification. A schematic diagram of its principle is shown in Fig. 6. B1–4 in the figure represent the four feature layers added to the attention mechanism of ResNet50. M1–4 layers represent the auxiliary loss of 4 characteristic layers, and P represents the main loss. The same supervision signal is added to the features of each layer. The specific steps are as follows:

#### 1. Consistency monitoring module

First, the feature pyramid is constructed based on the multiscale features (B1, B2, B3, B4) in the main feature box. The ROI characteristics of each level (M1, M2, M3, M4) are obtained through ROI align. Then, the ROI features of (M1, M2, M3, M4) are convoluted by 3 × 3 to obtain the feature pyramid (P1, P2, P3, P4) to generate multiple ROIs. A detector and a classifier are added after (P1, P2, P3, P4) each feature before fusion. These classification and regression parameters are shared at different levels, which can further force different feature maps to learn similar semantic information outside the same monitoring signal. For more stable optimization, the weight is used to balance the auxiliary loss and original loss caused by consistent supervision. Formally, the final loss function formula is as follows:

9 $$L_{rcnn} = \lambda \left[ {L_{cls,M} \left( {pM,t^{*} } \right) + \beta \left( {t^{*} > 0} \right)L_{loc,M} \left( {d_{M} ,b^{*} } \right)} \right] + L_{cls,P} \left( {p,t^{*} } \right) + \beta \left( {t^{*} > 0} \right)L_{loc,P} \left( {d,b^{*} } \right)$$

${L}_{cls,M}$ and ${L}_{loc,M}$ are the objective functions corresponding to the auxiliary losses attached to (M1, M2, M3, M4). ${L}_{cls,P}$ and ${L}_{loc,P}$ are the original loss functions on the characteristic pyramids (P1, P2, P3, P4). $pM$, ${d}_{M}$ and $p$, $d$ are the predictions of the middle layer and the final pyramid layer, respectively. ${t}^{*},{b}^{*}$ are basic fact category labels and regression targets, respectively. λ is the weight used to balance the auxiliary loss and the original loss. β is the weight used to balance classification and localization losses. Finally, these classification and regression parameters are shared at different levels, which can further force different features to map in the same monitoring. Learn similar semantic information outside the signal. As shown in Fig. 6 Schematic diagram of the AugFPN fusion framework (a), through the above measures, consistency monitoring can reduce the semantic gap between different scales of information.

#### 3. Residual feature enhancement

AugFPN fusion proposes residual feature enhancement to reduce the loss of semantic information caused by the reduction in the number of channels through spatial information compensation. First, B4 is downsampled into three parts as large as B4 through adaptive pooling. Then, the four layers are fused into P5. The weight of each layer is $\alpha 1,\alpha$2,$\alpha$3, which are 0.1, 0.2 and 0.3, respectively. After generating P5, it is combined with P4 by summation and propagated to other functions at a lower level. The residual feature enhancement structure is shown in Fig. 6b.

#### 3. Soft ROI feature selection

**Fig. 7:**
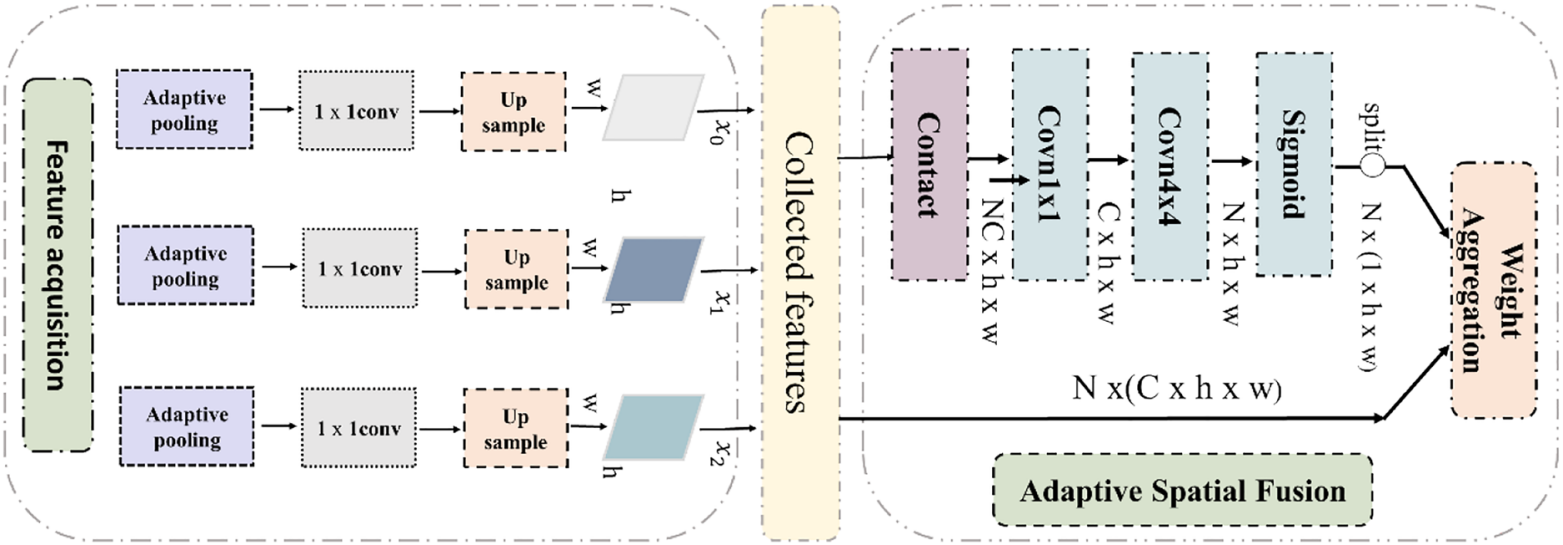
Fusion process of different levels of features and adaptive fusion framework diagram

First, because (P1, P2, P3, P4) each layer contains ROI features, we use the adaptive spatial fusion module (ASF) to adaptively fuse ROI features. ASF generates different spatial weight maps for different levels of region of interest features and weights and fuses the region of interest features. The specific fusion process of different levels of features and the framework of adaptive fusion (ASF) are shown in Fig. 7.

Based on the above principles, AugFPN reduces the semantic gap between different scale features before feature fusion through consistency monitoring. In feature fusion, the ratio invariant context information is extracted by residual feature enhancement to reduce the information loss of feature mapping at the highest pyramid level. The soft ROI selection method is used to better realize feature extraction and fusion through adaptive spatial fusion. Then, they are integrated with the full connection layer of the network, which can effectively solve the common problem of losing small image features in the main feature frame.

### Mixed activation function—ELU function

The introduction of an activation function increases the nonlinearity of the neural network model. The nonlinear expression ability of the activation function is strong. When the linear input is large, the output will not expand infinitely, which does not easily lead to gradient explosion. In addition, gradient descent can be effectively realized because the nonlinear activation function is differentiable. Traditional saturation activation functions such as sigmoid and tanh have the problem of gradient disappearance. This will make the convergence of the training network increasingly slower.

The activation function used in the original ResNet50 model is the ReLU [31] function. The linear and unsaturated form of the ReLU function allows the ResNet50 model to solve the problem of gradient disappearance in the positive region. However, if the input distribution after network initialization is not ideal, or a large gradient suddenly occurs in the training process, which affects the distribution of the next input, the distribution centre becomes negative. Then, most of the inputs in the ResNet50 model are negative. When the negative input is activated and zeroed, the gradient will not be obtained. Finally, the weight of the negative input cannot be updated.

**Fig. 8:**
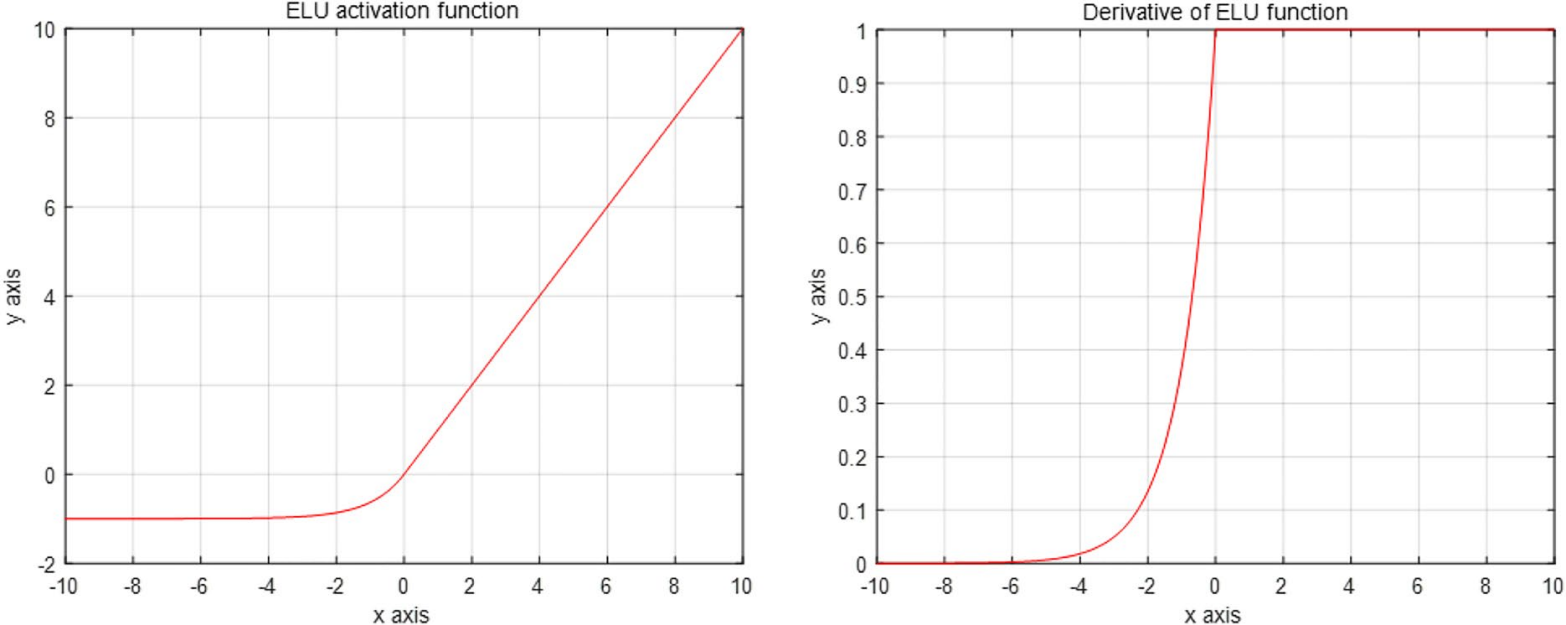
Image of the ELU function and its derivative function

In view of the above shortcomings of the ReLU function, we choose another improved ReLU-ELU activation function. The image and its derivative function are shown in Fig. 8:

The expression of the ELU function is:

10 $$f( x ) = \left\{ \begin{array}{ll} x, &\quad if\left( {x > 0} \right) \hfill \\ \alpha \left( {e^{x} - 1} \right), &\quad otherwise \hfill \\ \end{array} \right.$$

The ELU function is consistent with the part where the ReLU function is greater than 0. When it is less than 0, the ELU function expression is $\alpha ({e}^{x}-1)$. Thus, the ELU function still outputs when the input is negative. First, this ensures that the ELU function can inherit the advantages of the ReLU function and solve the problems of gradient explosion and gradient disappearance in the network. Second, when the input value of the ELU function is less than 0, the parameters can also be updated, which can effectively solve the problem of neuron death so that the negative part of the activation function can be used effectively. It can also make the network convergence faster.

### Label smoothing regularization

In neural network, because of too many model parameters, it is easy to cause overfitting of the model. Typical regularization methods such as L1, L2 and dropout [32] are used to suppress the overfitting phenomenon of the network due to too many model parameters. However, in citrus disease classification, we add the softmax function to calculate the probability that the input image belongs to each disease. Then, the image with the highest probability is used as the input of the disease category, and the cross entropy is used as the loss function. This leads to the maximum reward for correct classification and the maximum penalty for incorrect classification. Therefore, in the classification task, the phenomenon of overfitting easily exists. Therefore, in the MF-RANet network, this paper uses label smoothing regularization to alleviate the overfitting phenomenon in classification. The specific steps of label smoothing regularization are as follows:

In the citrus disease classification task, the confidence scores of citrus disease images corresponding to various diseases are obtained through the MF-RANet network. These scores are normalized by the softmax function [33], and finally, the probability that the current input belongs to each category is obtained. The formula of the softmax function is as follows, where k represents a total of 6 citrus images (5 disease images and 1 health image):

11 $${\text{q}}_{i} = \frac{{\exp \,\left( {z_{i} } \right)}}{{\mathop \sum \nolimits_{j = 0}^{K} {\text{exp}}\left( {z_{i} } \right)}}$$

Label smoothing changes the probability distribution into simple uniformly distributed noise. The formula is as follows: $\varepsilon$ varepsilon is a small super parameter:

12 $${\text{P}}_{\text{i}} = \left\{ \begin{array}{ll} (1 - \varepsilon ), &\quad f( i = y) \\ \frac{\varepsilon }{K - 1} &\quad if \ ( i \ne y ) \end{array} \right.$$

The cross entropy is

13 $${\text{Loss}}_{{\text{ i}}} = \left\{ \begin{array}{ll} (1 - \varepsilon )*{\text{Loss}}, &\quad if\left( {i = y} \right) \\ \varepsilon *{\text{Loss}} , &\quad if \ ( i \ne y) \\ \end{array} \right.$$

When training the MF-RANet network, the cross entropy of the prediction probability and label real probability is minimized to obtain the optimal prediction probability distribution. The prediction probability distribution of the optimal fitting effect of label smoothing is:

14 $${\text{Z}}_{i} = \left\{ \begin{array}{ll} \log \frac{{\left( {k - 1} \right)\left( {1 - \varepsilon } \right)}}{{\varepsilon + \alpha }}, &\quad if\left( {i = y} \right) \\ \alpha ,&\quad if\left( {i \ne y} \right) \\ \end{array} \right. \left( {\alpha \ {\text{can be any real number}}} \right)$$

The essence of label smoothing regularization is to suppress the output difference between positive and negative samples and smooth the label. The smoothed label can prevent the network from overlearning. This can effectively alleviate the overfitting phenomenon.

ResNet50 is the basic network of the MF-RANe network. Before the hidden layer of the ResNet50 network, there is a batch normalization layer to normalize the data. Therefore, the overfitting phenomenon of the network in training citrus disease samples is inhibited by batch normalization. Unlike batch normalization regularization, the use of label smoothing regularization can effectively alleviate the overfitting phenomenon in the classification process.

## Results

To analyse the effectiveness of the MF-RANet network in citrus disease identification and classification. We design experiments to compare the effectiveness of different models. However, because there are no clear standards and instructions for the specific codes and data segmentation of other models, we have to reproduce their models independently and conduct comparative experiments on the datasets we collected. In the comparative experiment, the test sets of different models are completely consistent. This part includes the experimental environment, experimental device, effectiveness analysis of each module of the MF-RANet network, evaluation index, ablation experiment and comparison experiment between different models.

## Laboratory environment

This paper constructs a neural network framework on Colaboratory, edits, compiles and runs the MF-RANet network on Colaboratory. The programming environment of the code is Python 3.7 and PyTorch. In addition, we use MATLAB to perform AMSR image enhancement. The hardware environment of the simulation experiment is a Google cloud disk GPU and Windows 10 (64 bit), in which the system memory is 32 GB.

## Experimental settings

The homemade dataset used in this paper contains 6 categories of citrus: Huanglong disease, Corynespora blight of citrus, fat spot macular disease, citrus scab, citrus canker and healthy citrus. The size of the input image is 224*224. This can improve the efficiency of image processing technology and reduce the time for model training and classification. After image enhancement and image amplification, we obtained a total of 10,100 citrus images. The number of six types of images is evenly distributed, all in the range of 15–20%. The dataset in this paper is divided into a training set: verification set: test set = 6:2:2. There are 6060 citrus images in the training set. There are 2020 citrus images in the test set.

In deep learning training, the selection of super parameters is difficult and time-consuming. This is because the best combination of super parameters depends not only on the model itself but also on the software and hardware environment. The super parameters of the MF-RANet network in this paper are shown in Table 2. Parameter Setting The Adam optimizer [34] is used in the model in this paper. The batch size of the experiment is set to 32, the momentum parameter is set to 0.9, and the epoch number is set to 200. Each round has 190 iterations, 200 epochs have 3200 iterations in total, and they are verified once every 1000 iterations. The weight attenuation value is $1\times {10}^{-4}.$ The initial learning rate of the first 20 epochs is set to 0.001, and the initial learning rate of the last 10 epochs is set to 0.005 to improve the fitting speed.

**Table 2:** Parameter Setting

Parameter category	Parameter name	Parameter setting
Adam	Learning rate(0–20)	0.001
	Learning rate(21–30)	0.005
	Weight decay	1 × 10^–4^
	Momentum	0.9
Input data parameters	Size of input images	(224,224)
	Batchsize	32
	Iteration epochs	200
	Iteration number	3200

### Effectiveness of AMSR

**Fig. 9:**
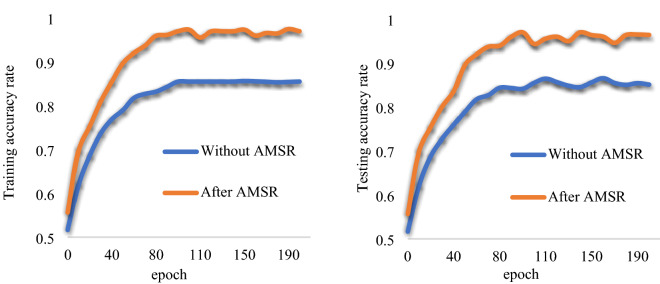
MF-RANet's citrus disease training and test accuracy images in the AMSR-enhanced dataset and non-AMSR-enhanced dataset

To verify the impact of the dataset after AMSR image enhancement on the classification performance, we use the MF-RANet network to train the dataset with image enhancement and the dataset without image enhancement simultaneously. The image divisions in the training set, the dataset verification set and the test set with and without image enhancement are the same. When other configurations are the same, the experimental results are shown in Fig. 9**.**

It can be seen in Fig. 9 that in the training set without AMSR image enhancement, when the epoch is 75 times, the MF-RANet network converges, and the final training accuracy is 85.41%. After the training set of AMSR image enhancement, when the epoch is approximately 75 times, the MF-RANet network converges, and the final training accuracy is 97.95% (+ 12.54%). Through the test, the final test accuracy obtained in the test set without AMSR image enhancement is 84.69%. The final test accuracy obtained in the test set enhanced by the AMSR image is 96.23% (+ 11.54%). Although the epoch times of MF-RANet convergence in the two training sets are the same due to the same training model, the recognition and classification accuracy of the MF-RANet network enhanced by the AMSR image is significantly higher.

Therefore, after the dataset is enhanced by AMSR, the training accuracy of the MF-RANet network is significantly improved. This shows that the AMSR algorithm makes the disturbed lesion features clearer after image enhancement. The clearer lesion features are easier to learn and recognize by the MF-RANet network.

### Performance comparison experiment between the ELU activation function and other activation functions

To verify that among the different activation functions, the ELU activation function is the most helpful for improving the recognition rate of citrus diseases in the MF-RANet network. We designed a comparative experiment of the influence of 5 activation functions on the network recognition rate and recorded the experimental results. The 5 activation functions selected in the comparative experiment are the tanh function [35], sigmoid function [36], ReLU function, leaky ReLU function [37] and ELU function. The tanh function and sigmoid function are traditional saturation activation functions, and the ReLU function is the activation function used by the original ResNet50 model. The leaky ReLU function and ELU function are activation functions improved based on the ReLU function. Both can be output in the interval less than 0, but the expression is different. First, this paper defines five networks with different activation functions but the same structure in the network: MF-RANet-1 (tanh), MF-RANet-2 (sigmoid), MF-RANet-3 (ReLU), MF-RANet-4 (leaky ReLU) and MF-RANet (ELU). This paper trains the above five networks with different activation functions but the same structure in the network with the same dataset. We record the citrus recognition accuracy and convergence time of the above five networks in the training set and test set in Table 3 Training accuracy and test accuracy using different activation functions in MF-RANet.

**Table 3:** Training accuracy and test accuracy using different activation functions in MF-RANet

Methods	Training accuracy (%)	Test accuracy (%)	Training time
MF-RANet-1(tanh)	76.70	75.41	6 h 21 min 05 s
MF-RANet-2(sigmoid)	79.73	79.06	6 h 15 min 41 s
MF-RANet-3(ReLU)	93.10	92.49	4 h 45 min 53 s
MF-RANet-4(leaky ReLU)	95.54	94.13	4 h 17 min 30 s
MF-RANet (ELU)	97.95	96.87	3 h 47 min 46 s

The data in Table 3 Training accuracy and test accuracy using different activation functions in MF-RANet show that each activation function has different effects on the MF-RANet network. When there are saturated tanh and sigmoid activation functions in the MF-RANet network, the training accuracy and testing accuracy of citrus diseases are greatly reduced, and the training time is greatly prolonged. This is because the phenomenon of gradient disappearance and gradient explosion under the action of the saturation activation function not only leads to the nonupdating of network parameters and reduce the recognition ability of the network but also greatly reduces the network training speed. In addition, according to our experimental data, the MF-RANet network significantly improves the recognition accuracy and training speed under the activation of leaky ReLU and ELU functions, but the effect of the ELU function is better. It is proven that the activation effect of the ELU function on the MF-RANet network is more obvious.

### Self-contrast experiment

To verify the performance of the improved MF-RANet network, we used the original unmodified ResNet50 and the improved MF-RANet to experiment on the same training set, verification set and test set of AMSR in the same test environment. Table 4 shows the performance comparison between ResNet50 and MF-RANet.

**Table 4:** Performance comparison of ResNet50 and MF-RANet

Method	ResNet50	MF-RANet
Training accuracy	85.74%	97.95%
Testing accuracy	85.06%	96.87%
mAP	79.49%	90.27%
FPS	49	88
GFLOPs	4.12G	6.1G
Training time	5 h 48 min 12 s	3 h 47 min 46 s
Model size (MB)	26 M	24 M

Table 4 Performance comparison of ResNet50 and MF-RANetshows that the training accuracy and test accuracy of the improved MF-RANet network are improved (+ 12.21%, + 11.81%). In terms of program running time, the training time of ResNet50 is 5 h 48 min 12 s, while the training time of the improved MF-RANet network is 3 h 47 min 46 s. The total training time was shortened by nearly 2 h. This reflects that the improved model has better performance in terms of training cost. In addition, the number of parameters of our improved MF-RANet network is much smaller than that before (− 2 M). In summary, the performance of our improved model is greatly improved compared with that of the basic model. Its effectiveness is reflected not only in preventing overfitting to improve the test accuracy but also in the time cost of training, which is very practical.

### Ablation experiment

To verify the effectiveness of each part of the MF-RANet network, we performed ablation experiments on the citrus image dataset enhanced by AMSR images. Taking ResNet50 as the backbone, RAM (R), AugFPN (a) the ELU activation function (E) (replace the activation function in the original ResNet50 with the ELU function as ReLU) and label smoothing regularization (L) are added. By comparing the test accuracy of the network for each kind of disease and the training time of the network, the performance of each module is analysed. The overall ablation experiment is shown in Table 5.

**Table 5:** Ablation Experiment

Network	Normal citrus (%)	Huanglong disease (%)	Corynespora blight of citrus (%)	Fat spot macular disease (%)	Citrus scab (%)	Citrus canker (%)	Average testing accuracy (%)	Training time	Model size (MB)
ResNet50	86.24	84.26	86.65	86.27	83.70	83.24	85.06	5:48ʹ12ʺ	26 M
ResNet50(E)	90.76	89.67	88.19	87.40	87.65	86.37	88.34	4:53ʹ40ʺ	26 M
ResNet50 + L	89.47	89.39	89.02	86.57	86.31	87.48	88.04	4:48ʹ33ʺ	26 M
ResNet50 + R	93.21	92.10	92.76	91.83	90.96	91.00	91.82	5:31ʹ10ʺ	25.2 M
ResNet50 + A	92.99	92.04	92.71	92.26	90.29	91.65	91.99	5:29 ʹ04ʺ	24.8 M
MF-RANet	97.55	96.77	96.22	95.96	93.93	94.04	96.87	3:47ʹ46ʺ	24 M

As seen from Table 5, the recognition rate of citrus diseases based on ResNet50 is the lowest among the six models. The average citrus test accuracy of the ResNet50 model increased (+ 3.28%, + 2.98%, + 6.77%, + 6.93%), and the training time decreased after adding the ELU activation function, label smoothing regularization, RAM and AugFPN (− 54ʹ38ʺ, − 59ʹ39ʺ, − 17ʹ02ʺ, − 19ʹ08ʺ). The size of the model is reduced (− 0 M, − 0 M, − 0.8 M, − 1.2 M). According to the rising data, the contribution of the four parts to the recognition rate in the MF-RANet network is AugFPN > RAM > ELU > label smoothing. Because ELU can inhibit gradient disappearance and gradient explosion, label smoothing can inhibit overfitting, and AugFPN and RAM can reduce parameters, the training time of the four models is shorter than ResNet50.

Compared with the recognition accuracy of 6 kinds of citrus images in the MF-RANet network, the recognition rate of healthy citrus images is the highest. The image recognition rates of the MF-RANet network for citrus canker and citrus scab are similar and lower than those of other diseases. This is mainly due to the high similarity of disease texture features between citrus canker and citrus scab. However, the recognition accuracy of the MF-RANet network for these 2 diseases is still higher than that of the other networks in the table for citrus canker and citrus scab. It still shows that the detailed feature frame designed in this paper has an effect on the identification of these 2 diseases with high similarity and improves the identification degree of similar features.

### Comparative experiment between the MF-RANet model and other networks

To compare the classification performance between the MF-RANet model and the neural network model. We compared the performance of eight models and tested the same dataset in the same experimental environment (the dataset has been enhanced by AMSR). During the experiment, we trained 8 networks for comparison from 0. Because there is no description of all codes and specific data of all networks, it creates repeatability problems. We have to reproduce most of these models independently and compare them when the datasets are completely consistent. We built the environment, reproduced the code, sorted out the dataset, trained the model and counted the training results we needed. The recognition accuracy, training time and model size of each model corresponding to each citrus image are filled in Table 6 The training and testing accuracy, training time, model size and GFLOPs of 8 models corresponding to citrus images. In this experiment, the experimental models we selected include the basic models MF-RANet, CNN, AlexNet [38], VGG16, and DesnseNet121 [39] and relatively new models, including NTS-Net [40], DFL-Net [41], and BSNet [42].

**Table 6:** The training and testing accuracy, training time, model size and GFLOPs of 8 models corresponding to citrus images

Network	Average training accuracy (%)	Average testing accuracy(%)	Training time	Model size (MB)	GFLOPs
CNN	65.84	68.36	10: 14ʹ57ʺ	0.6 MB	0.009
DenseNet121	83.74	82.39	7: 21ʹ55ʺ	14.15 MB	2.8
AlexNet	73.17	73.29	19: 34ʹ28ʺ	61 MB	0.7
VGG16	81.35	83.14	8: 00ʹ49ʺ	138 MB	15.5
NTS-Net	87.91	85.73	7: 38ʹ04ʺ	230 MB	17.81
DFL-Net	90.56	91.75	7: 04ʹ24ʺ	255 MB	18.6
BSNet	91.44	90.07	6: 51ʹ32ʺ	179 MB	15.03
MF-RANet	97.95	96.87	3: 47ʹ46ʺ	24 MB	6.1

Compared with the CNN, AlexNet, VGG16, DenseNet121, NTS-Net, DFL-Net, BSNet and MF-RANet models, the disease identification accuracy of the MF-RANet model proposed in this paper is generally higher than that of the other networks. This shows that the disease identification ability of the MF-RANet model built in this paper is higher than that of other common networks. Although the GFLOP values of VGG16, DenseNet121, NTS-Net, and DFL-Net are higher than those of the MF-RANet network, the final training time is much longer than that of the MF-RANet network because the network parameters of the above four models are too large. Thus, the value of the MF-RANet model in the current neural network model is verified.

### Performance verification experiment of the MF-RANet model under different datasets

To better verify the performance of the MF-RANett model, we found a total of 1,860 images of the same 6 kinds of citrus diseases on the PlantVillage [43] dataset. Among them, there were 285 images of citrus split skin disease, 313 images of citrus oil spots, 288 images of citrus sunburn, 305 images of citrus sand skin disease, 315 images of citrus black rot and 354 images of healthy citrus. The size of the input image is 224*224. After image enhancement and image amplification, we obtained a total of 7,440 citrus images. The dataset was divided into a training set: verification set: test set = 6:2:2. We used these 7740 images to carry out the supplementary experiment of citrus disease recognition.

**Fig. 10:**
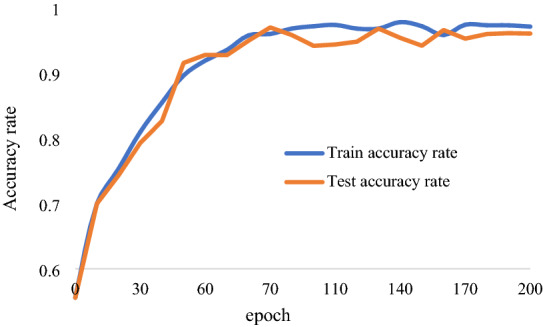
Recognition accuracy of citrus disease training and testing on the PlantVillage dataset

The experimental results are shown in Fig. 10. In Fig. 10, the curve in the figure shows the training and test recognition accuracy of six citrus images on the PlantVillage dataset. When epoch = 65, the training and testing accuracy curves converge and flatten. The highest training accuracy was 97.13%, and the highest test accuracy was 96.06%. This shows that our network structure can also achieve a better recognition effect on other kinds of citrus diseases in the PlantVillage dataset.

## Evaluating indicator

In fact, it is one-sided to only rely on classification accuracy to determine whether the model is truly effective. Therefore, this article selects the F1-score and its corresponding recall, precision and corresponding confusion matrix of 6 different citrus images as the evaluation index. The F1-score is the measurement function of precision and recall, and its calculation formula is as follows:

15 $${\mathrm{F}}_{1}=\frac{2\mathrm{PR}}{\mathrm{P}+\mathrm{R}}$$

16 $$\mathrm{P}=\frac{\mathrm{TP}}{\mathrm{TP}+\mathrm{FP}}$$

17 $$\mathrm{R}=\frac{\mathrm{TP}}{\mathrm{TP}+\mathrm{FN}}$$

In Formula (15), P represents precision, and R represents recall. The precision in the evaluation index is different from the accuracy of citrus recognition above in meaning and value. Precision in the evaluation index represents the average value obtained after calculating the correct prediction rate of each of the 6 disease prediction samples. TP in Formulas (16) and (17) represents the number of citrus disease samples predicted to be class A and actually class A. FP represents the number of citrus disease samples that are not predicted to be class a but are actually class A. FN represents the number of samples of citrus diseases predicted to be class a but not actually class A. The F1-score is an index used to measure the accuracy of the binary classification model in statistics. It takes into account both the accuracy and recall of the classification model. The F1-score can be regarded as a weighted average of model accuracy and recall. Its maximum value is 1, and its minimum value is 0. The greater the value is, the better the model performance.

In this experiment, recall, precision and F1-score are selected to verify the performance of the model. This article selects the above 12 models compared with MF-RANet and calculates their recall rate, precision and F1-score.

**Fig. 11:**
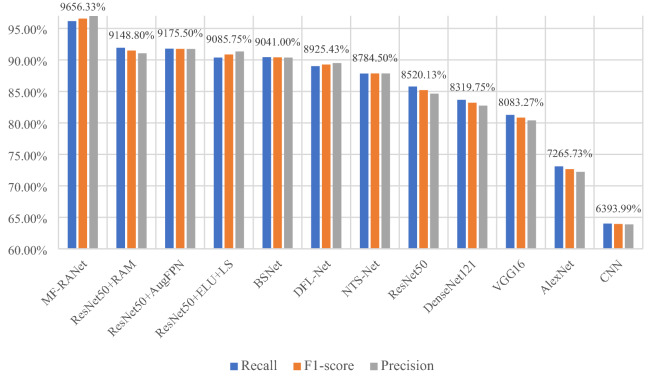
MF-RANet and other network model evaluation index histograms

Figure 11 MF-RANet is the accurate numerical histogram of the recall rate, precision and F1-score of the above 12 models. The data in the figure are F1-scores of 12 models obtained according to recall rate and precision. The experimental results show that the F1 value of the classification model in this article has reached the expected level of the experiment, which proves the correctness of the above discussion and analysis.

**Fig. 12:**
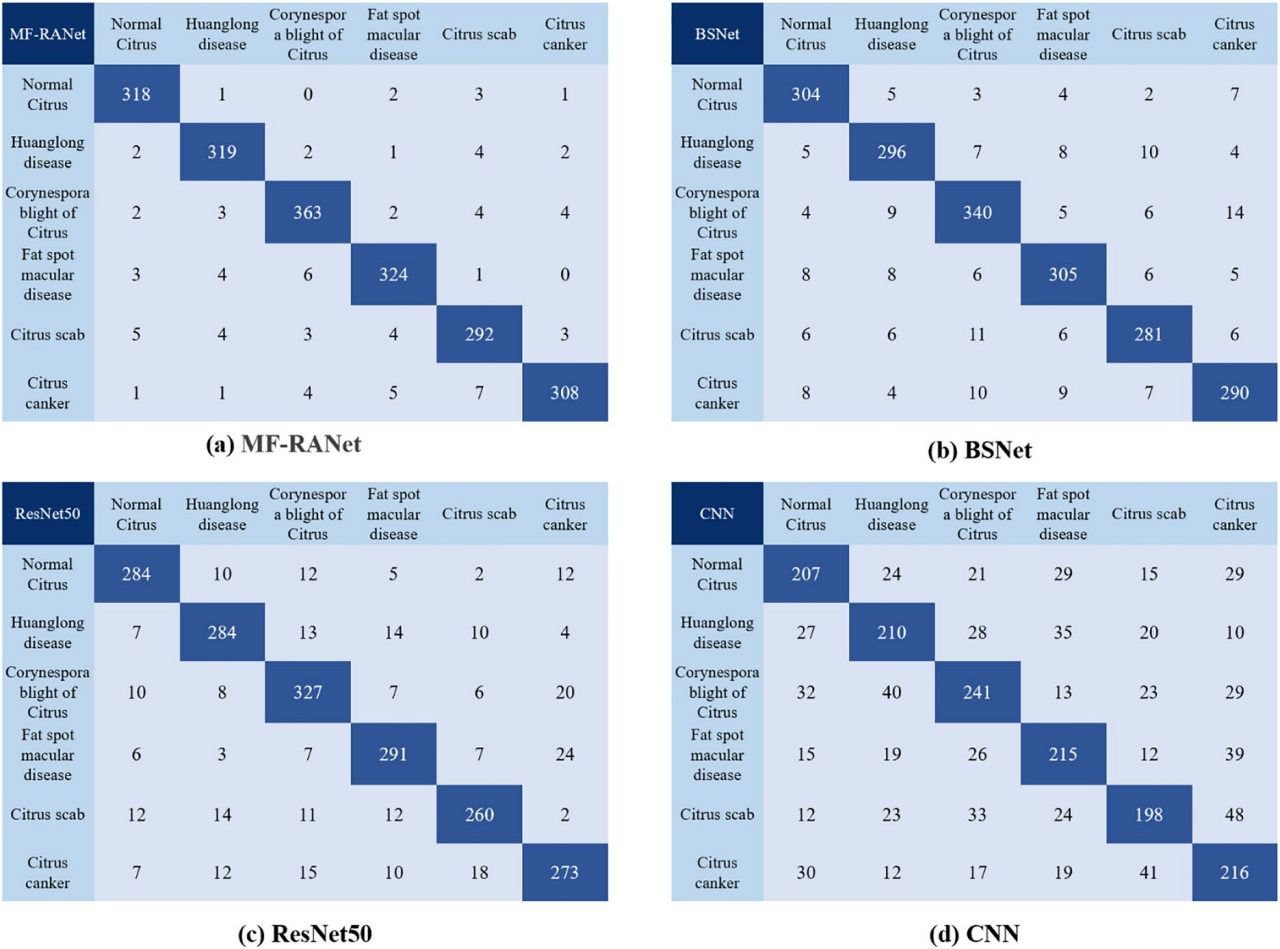
**a**–**d** Show the confusion matrices of MF-RANet, BSNet, ResNet50 and CNN, respectively

In the machine learning field, the confusion matrix is also called the possibility table or error matrix. It is a specific matrix used to visualize the performance of the algorithm, usually supervised learning (unsupervised learning, usually a matching matrix). Each column represents the predicted value, and each row represents the actual category. This is very important because in the actual classification, the TP value and FP value are the most direct indicators for determining whether the classification is correct. The F1-score value is the comprehensive embodiment of these two indicators. We selected and compared the confusion matrices of MF-RANet, BSNet, ResNet50, and CNN, as shown in Fig. 12a–d show the confusion matrices of MF-RANet, BSNet, ResNet50 and CNN, respectively.

From the confusion matrix of the above four models, it can be seen that the MF-RANe model proposed in this paper has a good image recognition effect of citrus diseases. Compared with the ResNet50 network, CNN network and BSNet, the FP values of citrus canker and citrus scab with a large number of complex and similar characteristics in the MF-RANet model are significantly lower than those of the other models. This is because the algorithm of the MF-RANet model tends to pay more attention to the main features and the features that retain the detailed features and filter out the useless information. The decrease in the FP value increases the recall rate of these 2 kinds of disease identification. According to Eq. (15), under the same precision, the increase in the recall rate eventually increases the F1 value. Therefore, experiments show that the MF-RANet model is more powerful in terms of integrity and has a good effect on improving the F1 value. In the same experimental environment, our algorithm is more suitable for citrus disease recognition than other classification models.

## Discussion

Through the above experiments, it can be seen that the AMSR preprocessing + MF-RANet network designed in this article has a good effect in realizing the identification of citrus diseases. However, the network designed in this article still has some defects, so the recognition rate of all types of diseases cannot reach 100%. AMSR preprocessing improves the retinex algorithm to achieve image enhancement, and the retinex algorithm also has a certain denoising effect. 2. Therefore, in AMSR preprocessing, the denoising process causes the loss of some features. Although there are detail feature frames in the MF-RANet network to reduce the loss of detail features, it can only reduce the details lost in the main feature frame. It cannot reduce the detailed features lost before entering the MF-RANet network. 2. Due to the imperfection of transmission media and recording equipment, digital images are often polluted by a variety of noise in the process of transmission and recording. Although the retinex algorithm in AMSR preprocessing can play a certain role in denoising, it cannot completely filter the noise, and the greater the noise generated by the picture is, the greater the impact on the recognition accuracy.

**Fig. 13:**
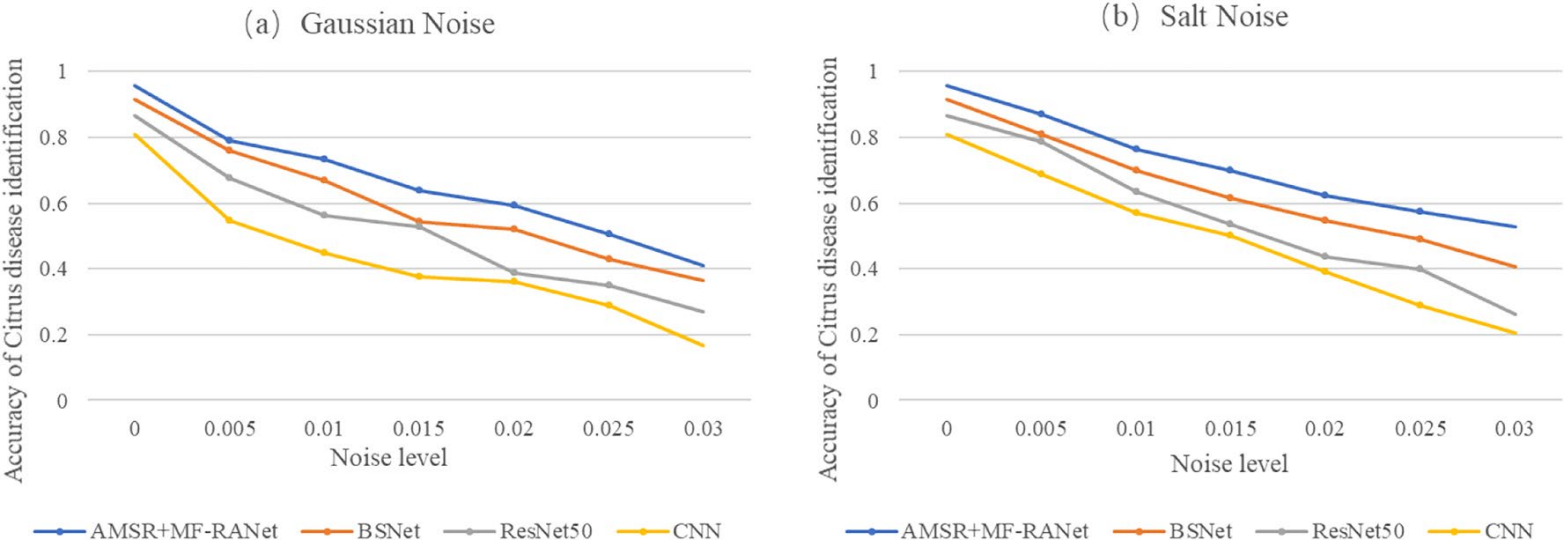
**a** Recognition accuracy of each network after adding Gaussian noise. **b** Recognition accuracy of each network after adding salt noise

Therefore, this article adds different levels of noise to the test set of the dataset collected in this article and selects four models, AMSR + MF-RANet, BSNet, ResNet50 and CNN, for experimental analysis. After adding salt noise and Gaussian noise, the accuracy of these models for citrus disease recognition is shown in Fig. 13.

Figure 13 shows that when two kinds of noise with different levels are added, the recognition accuracy of the AMSR preprocessing + MF-RANet network is the highest among the four methods as a whole. According to Fig. 13a and b, the recognition accuracy of the AMSR preprocessing + MF-RANet network decreases gradually with the improvement of the noise level. However, among the four different levels of noise interference, the change is the slowest. This shows that among the four networks, the AMSR preprocessing + MF-RANet network has the strongest anti-interference ability to noise. The reason is that the retinex algorithm in AMSR preprocessing also has a certain denoising function in image enhancement. However, because the enhancement function of AMSR preprocessing is significantly greater than the denoising function, the recognition rate decreases with the improvement of the noise level.

## Conclusions

With the progress of deep learning and computer vision technology, image recognition, image analysis and other fields have been widely studied. In recent years, crop disease image recognition and management based on deep learning have also been widely used. To improve citrus yield, ResNet50 was improved in this paper. From the test results of the improved model, we can see that.

1. To eliminate the interference of environmental factors such as low local illumination or shadows caused by clouds, dust and leaves, the AMSR enhancement algorithm designed in this paper can not only brighten the citrus disease image overall but also enhance the local illumination where the local illumination is low, make the features of the input image more obvious and reduce the difficulty of recognition.
2. The RAM and AugFPN feature fusion method is used to enhance the model parameters and improve the detection ability of the model. In the MF-RANet network, RAM used in the main feature frame and AugFPN used in the detail feature frame have a residual structure, which can effectively broaden the width of ResNet50. In this article, label smoothing regularization is used to improve the generalization ability of the whole network. Additionally, the ELU function is selected to improve the anti-interference ability of the network.
3. Through the comparison of MF-RANet and many other models, it is found that the citrus disease recognition effect of MF-RANet combined with AMSR pretreatment is the best in the selected network, and the recognition rate of different kinds of citrus diseases is the highest.
4. According to the experimental results in Fig. 10 Recognition accuracy of citrus disease training and testing on the PlantVillage dataset, when MF-RANet is applied to the PlantVillage dataset, the accuracy of citrus disease identification can also be effectively improved by applying MF-RANet to other datasets. This is of great significance to the application level of citrus disease identification. Compared with the traditional ResNet50 model, the MF-RANet network designed in this paper can not only greatly reduce the number of parameters and improve the operation efficiency but also improve its recognition accuracy. This can also reduce the occupation of hardware resources and verify the effectiveness of the model.

In life, the network designed in this article is beneficial for fruit farmers to accurately control the disease situation of citrus orchards. For example, some diseases are not easily detected by human beings. Through machine identification, the spread of diseases can be strangled at the onset in time to reduce economic losses as much as possible. In addition, the model can also play an important role in foreign trade exports and economic growth. In the follow-up, the model will be tested in real life, improved and applied to society as soon as possible to contribute to the economic production of citrus.

## Data Availability

The data presented in this study are available on request from the corresponding author. The data are not publicly available due to partial author disagreement. We are also very grateful to Li Liujun for his translation and polishing support for this article.

## Abbreviations

AMSR: Adaptive multi-scale retinex
MF-RANet: Multiscale feature fusion residual attention network
RAM: Residual attention mechanism
AugFPN: Aug feature pyramid networks
ELU: E-xponential linear unit

## References

[1] Guo WW, Ye JL, Deng XX (2019). 70 years of scientific research on fruit trees in new China; citrus. J Fruit Sci.

[2] Xiao R, Su SL, Mai GC (2015). Quantifying determinants of cash crop expansion and their relative effects using logistic regression modelling and variance partitioning. Int J Appl Earth Obs Geoinf.

[3] Jiao He (2020). Present situation and control countermeasures of citrus yellow dragon disease in Lingshan county. Guangxi Plant Prot.

[4] Stegmayer G, Milone DH, Garran S (2013). Automatic recognition of quarantine citrus diseases. Expert Syst Appl.

[5] Zhang W, Hu J, Zhou G, He M (2020). Detection of apple defects based on the FCM-NPGA and a multivariate image analysis. IEEE Access.

[6] Zhang W, Tan A, Zhou G, Chen A, Hu Y (2021). A method for classifying citrus surface defects based on machine vision. J Food Meas Charact.

[7] Mohanty SP, Hughes DP, Salethe M (2016). Using deep learning for image-based plant disease detection. Front Plant Sci.

[8] Lin H, Lin H, Zhou G (2021). EM-ERNet for image-based banana disease recognition. J Food Meas Charact.

[9] Xu L, Lv J (2017). Recognition method for apple fruit based on SUSAN and PCNN. Multimed Tools Appl.

[10] Zhang SW, Shang YJ, Wang L (2021). Leaf image-based plant disease identification using color and texture features. Wireless Pers Commun.

[11] Zhou G, Zhang W, Chen A (2019). Rapid detection of rice disease based on FCM-KM and faster R-CNN fusion. IEEE Access.

[12] Chen X, Zhou G, Chen A, Yi J, Zhang W, Hu Y (2020). Identification of tomato leaf disease-s based on combination of ABCKBWTR and B-ARNet. Comput Electron Agric.

[13] Jobson DJ, Rahman Z, Woodell GA (2002). A multiscale retinex for bridging the gap between color images and the human observation of scenes. IEEE Trans Image Process.

[14] Sankaran S, Ehsani R, Etxeberria E (2010). Mid-infrared spectroscopy for detection of Huanglongbing (greening) in citrus leaves. Talanta.

[15] Liu J, Xuewei W (2020). Early recognition of tomato gray leaf spot disease based on MobileNetv2-YOLOv3 model. Plant Methods.

[16] Chen X, Zhou G, Chen A, Pu L, Chen W (2021). The fruit classification algorithm based on the multi-optimization convolutional neural network. Multimed Tools Appl.

[17] Lv M, Zhou G, He M (2020). Maize leaf disease identification based on feature enhancement and DMS-robust alexnet. IEEE Access.

[18] Luong M-T, Pham H, Manning CD. Effective approaches to attention-based neural machi-ne translationin. In: Proceedings of the Conference on Empirical Methods in Natural Language Processing; 2015.

[19] Cohn T, Vu Hoang CD, Vymolova E, Yao K, Dyer C, Haffari G. Incorporating structural alignment biases into an attentional neural translation model. In: Proceedings of NAACL-HLT; 2016.

[20] Tu Z, Lu Z, Liu Y, Liu X, Li H. Modelling coverage for neural machine translation. In: Proceedings of the 54th Annual Meeting of the Association for Computational Linguistics; 2016

[21] Hines GD, Rahman Z, Jobson DJ, Woodell GA. DSP implementation of the retinex image enhancement algorithm. Proc SPIE 5438, Visual information processing XIII; 2004. 10.1117/12.544500

[22] China Science Data Network, http://www.csdata.org/. 2021;10.

[23] Digipathos Website, https://www.digipathos-rep.cnptia.embrapa.br/. 2021;10.

[24] K He, X Zhang, S Ren and J Sun. Deep residual learning for image recognition. 2016 IEEE conference on computer vision and pattern recognition (CVPR); 2016, p. 770–778. 10.1109/CVPR.2016.90.

[25] Krizhevsky A, Sutskever I, Hinton GE. Imagenet classification with deep co-nvolutional neural networks[C]//PEREIRA F, BURGES C J C. The 25th International conference on neural information processing systems. Nevada American: MIT Press; 2012. p. 1097–1105.

[26] Girshick R, Donahue J, Darrell T, et al. Rich feature hierarchies for object detection and semantic segmentation[C]//IEEE conference on computer vision and pattern Recogn-ition(CVPR). Columbus: IEEE; 2014. p. 580–587.

[27] Girshick R. Fast R-CNN[C]//proceedings of IEEE international conference on computer vision. Los Alamitos: IEEE Computer Society Press; 2015. p. 1440–1448

[28] Ren S, He K, Girshick R (2017). Faster R-CNN: towards real time object detection with region proposal networks. IEEE Trans Pattern Anal Mach Intell.

[29] Ren S, He K, Girshick R, Sun J (2017). Faster R-CNN: towards real time object detection with region proposal networks. IEEE Trans Pattern Anal Mach Intell.

[30] Linty, Dollărp, Girshickr, et al. Feature pyramid net-works for object detection[C]//2017 IEEE conference on computer vision and pattern recognition (CVPR). Honolulu: IEEE; 2017. p. 936–944. 10.1109/CVPR.2017.106.

[31] Nair V, Hinton GE. Rectified linear units improve restricted boltzmann machines. In: Proceedings of international conference on machine learning. 2010. p. 807–814.

[32] Srivastava N, Hinton GE, Krizhevsky A, Sutskever I, Salakhutdinov R (2014). Dropout: a simple way to prevent neural networks from overfitting. J Mach Learn Res.

[33] Salakhutdinov R, Hinton G. Replicated Softmax: an undirected topic model. In: Proceedings of the 22nd International Conference on Neural Information Processing Systems (NIPS'09). New York: Curran Associates Inc.; 2009. p. 1607–1614.

[34] Yaguchi A, Suzuki T, Asano W, Nitta S, Sakata Y, Tanizawa A. Adam induces implicit weight sparsity in rectifier neural networks. In: Proceedings of the 17th IEEE international conference on machine learning and applications. 2018. p. 318–325.

[35] Lohani HK, Dhanalakshmi S, Hemalatha V, Mallick PK, Balas VE, Bhoi AK, Zobaa AF (2019). Performance analysis of extreme learning machine variants with varying intermediate nodes and different activation functions. Cognitive informatics and soft computing.

[36] Singh BV, Kumar V (2019). Linearized sigmoidal activation: a novel activation function with tractable non-linear characteristics to boost representation capability. Expert Syst Appl.

[37] Yisi L, Wang X, Wang L, Liu D (2019). A modified leaky ReLU scheme (MLRS) for topology optimization with multiple materials. Appl Math Comput.

[38] Krizhevsky A, Sutskever I, Hinton GE (2012). Imagenet classification with deep convolutional neural networks. Adv Neural Inf Process Syst.

[39] Huang G, Liu Z, Van Der Maaten L, Weinberger KQ. Densely connected convolutional networks. In: Proceedings of the IEEE Conference on computer vision and pattern recognition. Honolulu; 2017. p. 4700–4708.

[40] Yang Z, Luo T, Wang D, Wang D, Hu Z, Gao J, Wang L, Ferrari V, Hebert M, Sminchisescu C, Weiss Y (2018). Learning to navigate for fine-grained classification. European conference on computer vision.

[41] Wang Y, Morariu VI, Davis LS. Learning a discriminative filter bank within a CNN for fine-grained recognition. In: Proceedings of the 2018 IEEE/CVF conference on computer vision and pattern recognition. Salt Lake City; 2018. p. 4148–4157.

[42] Li X, Wu J, Sun Z, Ma Z, Cao J, Xue JH (2021). BSNet: Bi-similarity network for few-shot fine-grained image classification. IEEE Trans Image Process.

[43] Hughes DP, Salathe M. An open access repository of images on plant health to enable the development of mobile disease diagnostics. arXiv: Computers and society; 2015.

